# Network robustness and structure depend on the phenological characteristics of plants and pollinators

**DOI:** 10.1002/ece3.8055

**Published:** 2021-09-10

**Authors:** Laura Melissa Guzman, Scott A. Chamberlain, Elizabeth Elle

**Affiliations:** ^1^ Evolutionary and Behavioural Ecology Research Group Department of Biological Sciences Simon Fraser University Burnaby British Columbia Canada

**Keywords:** mutualism, network, phenology, plant–pollinator, trait

## Abstract

Many structural patterns have been found to be important for the stability and robustness of mutualistic plant–pollinator networks. These structural patterns are impacted by a suite of variables, including species traits, species abundances, their spatial configuration, and their phylogenetic history. Here, we consider a specific trait: phenology, or the timing of life history events. We expect that timing and duration of activity of pollinators, or of flowering in plants, could greatly affect the species' roles within networks in which they are embedded. Using plant–pollinator networks from 33 sites in southern British Columbia, Canada, we asked (a) how phenological species traits, specifically timing of first appearance in the network and duration of activity in a network, were related to species' roles within a network, and (b) how those traits affected network robustness to phenologically biased species loss. We found that long duration of activity increased connection within modules for both pollinators and plants and among modules for plants. We also found that date of first appearance was positively related to interaction strength asymmetry in plants but negatively related to pollinators. Networks were generally more robust to the loss of pollinators than plants, and robustness increased if the models allow new interactions to form when old ones are lost, constrained by overlapping phenology of plants and pollinators. Robustness declined with the loss of late‐flowering plants, which tended to have higher interaction strength asymmetry. In addition, robustness declined with loss of early‐flying or long‐duration pollinators. These pollinators tended to be among‐module connectors. Our results point to networks being limited by early‐flying pollinators. If plants flower earlier due to climate change, plant fitness may decline as they will depend on early emerging pollinators, unless pollinators also emerge earlier.

## Graphical abstract

In plant–pollinator networks of southern British Columbia, we found that networks were generally more robust to the loss of pollinators than plants. Robustness declined with the loss of late‐flowering plants, and with the loss of early‐flying or long‐duration pollinators.

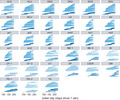

## 1 INTRODUCTION

Species within communities form interaction networks. Many species attributes contribute to their roles within a network, including traits like flower color and feeding preferences (Valdovinos, 2019; Vázquez et al., 2009), abundance (Valdovinos, 2019; Vázquez et al., 2007), spatial configuration (Morales & Vázquez, 2008), phylogenetic history (Chamberlain et al., 2014; Rezende et al., 2007), and phenology (the timing of life history events, Peralta et al., 2020). These traits can determine why some species interact with one another, with a key hypothesis being trait matching: Species interact when their traits make that interaction possible (e.g., Eklöf et al., 2013; Jordano, Bascompte, & Olesen, 2003; Pichler et al., 2020). Matching of phenologies is essential for determining individual species interactions and thus networks as a whole. For example, flowering timing depends on pollination syndrome which effectively determines the species likely to pollinate a given plant (Cortés‐Flores et al., 2017), and webs formed via phenological processes have a distinct structure from webs created by neutral processes (Simmons et al., 2020). Species phenology has been shown to affect network robustness (the ability to withstand perturbations) (Encinas‐Viso et al., 2012, Ramos‐Jiliberto et al., 2018, CaraDonna et al., 2014; CaraDonna et al., 2017; Vizentin‐Bugoni et al., 2020).

Phenology is important to consider in the context of perhaps the biggest perturbation communities will experience during the 21st century: climate change. We know that phenology shifts with climate (Bartomeus et al., 2011), and that different species may respond to climate by different magnitudes. For example, early‐season angiosperms may advance more than those that flower later in the season (Wolkovich et al., 2012). In addition, shifts in phenology by one species may result in phenological mismatch (loss of trait matching) with their interacting partner, unless both species respond similarly. Some studies show that both plants and pollinators shift their phenology at the same rate (Bartomeus et al., 2011; Hegland et al., 2009), but there is considerable variation among studies, and in some systems, mismatches have been observed (e.g., Gordo & Sanz, 2005). These system‐dependent results point to the complexity of responses, some of which may have consequences for networks. Given the rapid pace of climate change and its global scale, we need to understand how the phenology of specific species determines their role within a network, and the potential impact of shifting species phenologies on network robustness. Specifically, we explored two variables that can describe the phenology of a species: when a species emerges or flowers for the first time during a season, and how long a species is active during the season. Shifts in the timing of both of these variables can lead to mismatches with potential interaction partners (Hegland et al., 2009), potentially affecting network robustness.

Robustness is the tolerance of a network to the removal of a species (Memmott et al., 2004) and is a useful metric for network stability. A number of network metrics are associated with robustness. For example, modules in a network—groups of species that interact more with one another—can be the result of habitat heterogeneity, co‐evolution or phylogenetic relatedness of the species, or species traits like phenology (Lewinsohn et al., 2006; Morente‐López et al., 2018; Pimm & Lawton, 1980; Thompson, 2005), and modularity is associated with the stability of networks (Thébault & Fontaine, 2010, Stouffer & Bascompte, 2011). In seed dispersal networks for example, plant and animal trait values—body mass and seed mass—were associated with the modularity of individual species (Donatti et al., 2011, where species modularity describes whether species are peripheral or highly connected both within or among modules). Other aspects of network structure that are important for robustness are the degree of specialization of species and the asymmetry of interactions (Kaiser‐Bunbury et al., 2010; Mello et al., 2011). Specialization measures how “evenly” species interact with their partners, and interaction asymmetry measures the mismatch between a focal species' effect on its interaction partners and the effect of the interaction partners on the focal species. Given the relationship between species phenological traits and network properties, and the relationship between network properties and network stability (Rohr et al., 2014; Song & Saavedra, 2018), we would expect that phenological traits themselves would be one of the mechanisms that underlie the relationship between network structure and network stability. Indeed, Encinas‐Viso et al. (2012) and Ramos‐Jiliberto et al. (2018) used dynamical models to study the effect of phenological traits on the stability and robustness of networks. Encinas‐Viso et al. (2012) found that as the length of the season of activity increases, richness and resilience of the network also increase. Ramos‐Jiliberto et al. (2018) went a step further and combined dynamical models with empirical networks, and found that the loss of plants with earlier blooming dates and with longer active periods decreased pollinator persistence. While these studies used dynamical models, here we use empirical data to test the relationship between phenological traits and species roles in a network, as well as the relationship between phenological traits and network robustness.

We used 33 mutualistic plant–pollinator interaction networks from Western Canada to ask how plant and pollinator phenology contribute to their network interaction structure. We focus on exploring four measures of network structure that are known to be related to robustness: specialization, within‐module degree, among‐module connectivity, and interaction asymmetry. Specifically, we ask the following two questions: (a) How do date of first appearance in a network, and length of activity during the season, affect individual species interaction patterns? We predict that species whose date of first appearance is earlier, and that are active longer in the season, should be less specialized, have greater within‐module degree, greater among‐module connectivity, and have higher values of interaction asymmetry (they affect their partners more than the reverse). (b) How robust are networks to removal of species due to varying phenological “traits” (date of first appearance early/late, duration of activity short/long)? Networks should be more robust to losing species whose date of first appearance is later in the season, and species that are active during less of the season.

## 2 METHODS

### 2.1 Study sites

A total of 33 mutualistic plant–pollinator networks were studied in British Columbia, Canada: oak savanna (12 networks), shrub–steppe (eight networks), and restored hedgerows (13 networks). These three vegetation types comprised three different studies. See Table S1 and Figure S1 for site information, including latitude/longitude coordinates. The average distance between sites within studies was 19, 18, and 29 km for the oak savanna, shrub–steppe, and hedgerows, respectively. For simplicity, we use “pollinator” throughout this paper to refer to insects and hummingbirds observed visiting flowers and contacting reproductive organs, although their effectiveness at transfer of pollen has not been assessed. The networks were comprised largely of bees, with wasps and hoverflies also common. Less common were butterflies and beetles. The plants were largely forbs with some shrubs; insect‐pollinated trees were not sampled for largely logistical reasons of tree height but tended to be uncommon in these ecosystems.

### 2.2 Collection of mutualistic network data

Data were collected for two of three vegetation types using the plot method and for the third using the transect method. Plots are generally more appropriate when the plant species in the community are very patchily distributed (Gibson et al., 2011), as they were in these regions. The plot method focuses on individual plant species, observing each plant species for an equal amount of time. For oak savanna sites (within the Coastal Douglas Fir biogeoclimatic zone), we collected data on species interactions in 1‐ha plots at each of six sites in both 2009 and 2010, resulting in 12 networks. Each plot was surveyed about every 7–10 days, 10–12 times per season between late April and early July, the majority of the flowering period. Over the flowering period, we attempted to visit sites morning, midday, and afternoon on different survey dates to reduce bias due to flight time differences among visiting insects. During each survey date, each plant species in flower was observed for a 10‐min period by each of two surveyors, on haphazard walks throughout the plot. All flower visitors were collected and identified to the lowest taxonomic level possible. For the eight shrub–steppe sites (in the Bunchgrass biogeoclimatic zone), data were collected as for oak savanna sites, but surveys were from the beginning of April through the end of July, 2010, for a total of 12 samples per site. These sites were only sampled in 1 year (2010) and resulted in eight networks.

For the restored hedgerows sites, data were collected using a “transect” method, in which the plants along the transect were observed for a set amount of time, with time observed per plant species varying among species depending on their occurrence in the transect. Transects are more appropriate when plants are not clumped, but are widely scattered throughout a study site (Gibson et al., 2011), and in this case, most of the restorations (within the coastal Western Hemlock biogeoclimatic zone) were linear, making transects efficient. Sampling was equal across all 13 sites, occurring approximately every 2 weeks, for a total of nine samples between late April and the end of August, 2013. Hedgerow sampling resulted in 13 networks from 13 sites where each network was comprised of nine samples. The transect was walked for 15 min by each of two observers during each sample date, and each site was again observed equally during morning, midday, and afternoon on different sample dates. Once again, all flower visitors were collected and identified to the lowest taxonomic level possible.

### 2.3 Species phenological variables

**FIGURE 1:**
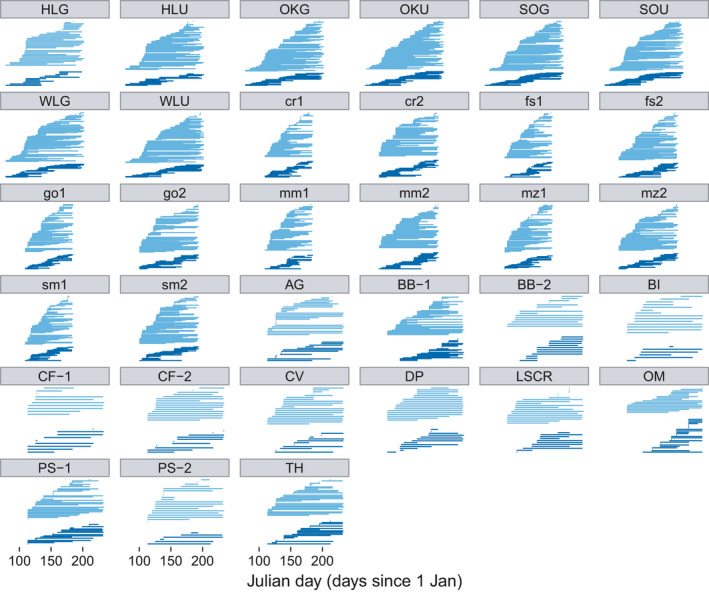
Visualization of the phenology of species in each network, for all 33 networks. The top set of horizontal lines in each panel are pollinators, and the bottom set are plants. Note that each panel follows the same *x*‐axis

We collected the following phenological species variables for every pollinator and plant species in each network: (a) first Julian day observed interacting in the network, and (b) number of days observed in the network (last date observed–first date observed). We treat each of the 33 networks as replicates and calculate both phenological variables for all species within each network. This means that there is no single value for first day observed or total days observed for any particular species across networks. Phenological variables were calculated from the observation of the interactions (Figure 1). While we acknowledge that calculating phenology from the observation of the interactions is imperfect, it is consistent across all 33 networks and has been previously used (Rasmussen et al., 2013). The number of days and first Julian day were log_10_ transformed to improve assumptions of normality, checked through histograms and qq‐plots. Because these two phenological variables could potentially be correlated (they are calculated from the same set of data), we calculated Pearson's correlation coefficient for log10‐transformed variables for each of plants and pollinators separately. We found that the two variables were moderately to weakly correlated (pollinators: *ρ* = −0.5, *p* < 0.001, *df* = 1,690; plants: *ρ* = −0.25, *p* < 0.001, *df* = 590), meaning that if a species has a very late First Julian day it cannot, by definition, be present many days in the network. In contrast, if a species has an early First Julian day, it can be present many days, or few days.

### 2.4 Network metrics

Before calculating network metrics, we normalized network matrices by dividing each cell value by the number of days the community was observed (zeros remain zeros). We did this because there was unequal sampling effort across studies, even though there was equal effort across networks within studies. Normalizing resulted in noninteger values in some cases, but standardized individuals observed per unit time; therefore, the network is weighted by the frequency of interactions for a given monitoring time. This normalization allowed us to control for differences in sampling effort between studies but it did not control for differences in the ecosystems themselves. We account for this variation between ecosystems in the analyses as explained below.

We calculated four species‐level network properties: (a) standardized specialization for each species (*d*′) following Blüthgen et al. (2006). We used this measure instead of species degree (number of other species the focal species interacts with), because degree is based on a binary matrix, and so does not utilize information on the frequency of interactions. For example, degree does not take into account if a single plant represents 99% of all of the interactions with a given pollinator. Specialization (*d*′), on the other hand, takes into account how “evenly” species interact with their partners; therefore, for the same number of interacting partners, utilizing one partner 99% of the time versus as they are available will result in different levels of specialization. *d*′ is based on Shannon's entropy; therefore, it can be interpreted as the deviation from the null distribution of interaction frequencies that assumes that all partners are used in proportion to their availability. We also calculated (b) interaction strength asymmetry (ia), which measures the average mismatch between a focal species' effect on its interaction partners and the effect of the interaction partners on the focal species (Vázquez et al., 2007). The interaction strength is assumed to be proportional to the frequency of interaction between two species and takes into account all of the other interactions. As a result, the interaction strength asymmetry allows us to compare among networks of different sizes, as well as, the relative strength of all interacting species.

**FIGURE 2:**
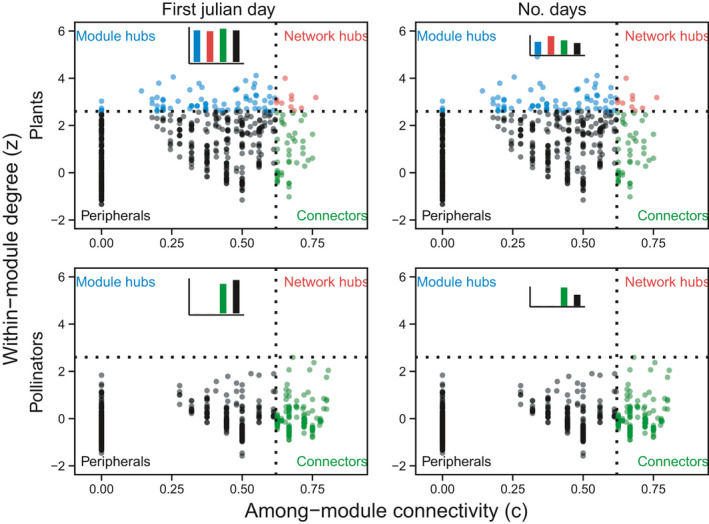
Visualization of species‐level modularity metrics of within‐module degree and among‐module connectivity for plants and pollinators. Each panel is split into four quadrants: Module hubs: species with high *z*, but low *c*, or those interacting a lot within their module, but not much among modules; Network hubs: species with both high *z* and *c*, or super generalists, acting as both connectors and module hubs; Connectors: species with low *z*, but high *c*, or those not interacting a lot within their module, but tending to connect modules; and Peripherals: species with both low z and c, or specialists, that is, they have only a few links and mostly within the module. Left‐hand panels: bar plots show mean values of first Julian day of appearance in the network for each quadrant. Right‐hand panels: bar plots show mean values of total number of days observed in the network for each quadrant. Format following Olesen et al. (2007)

In addition, for each network we calculated modularity and identified modules—sets of species that are more connected to each other than to other species in the network (Olesen et al., 2007)—and for each species we calculated (c) within‐module degree (*z*, the standardized number of links per species within a module), and (d) among‐module connectivity (*c*, how well does the species connect different modules). We chose these because we were interested in how phenology affected network metrics, so needed to use metrics that were quantified at the species level (where phenology was varying). In addition, within‐module degree (*z*) and among‐module connectivity (*c*) characterize the roles that species play in a network, providing a rich way of understanding networks (Olesen et al., 2007, see also Figure 2). We found that 27 out of the 33 networks had significant modularity (Table S1).

We used the *specieslevel* function in the bipartite R package (Dormann, 2009) to calculate *d*′, and ia (interaction push‐pull in specieslevel). To calculate the species‐level modularity metrics (*z* and *c*), we used a modularity‐detecting algorithm, which maximized modularity using simulated annealing (SA) implemented in the command line function *netcarto_cl* in the C program Rgraph (Guimerà & Amaral, 2005a, 2005b). The parameters we used for *netcarto_cl* were interaction factor: 0.8, cooling factor: 0.99, and randomizations: 100. All other analyses were done with the programming language R version 3.6.2 (R core team, 2019).

### 2.5 Data analyses

#### 2.5.1 Network structure and phenology analysis

We tested for a relationship between the four species‐level network structures (*d*′, ia, *c, z*) and phenological variables using lme4, lme4Test (Bates et al., 2015; Kuznetsova et al., 2017). For ia and *z* (as continuous values from 0 to infinity), we used linear mixed effects models, with two phenology variables (date of first appearance and days observed) as fixed effects and network nested within study as a random effect (1|study/network). We also included a random effect for taxonomic group at the family level. This random effect was included as some groups will tend to have a longer duration in the season; for example, bumble bees have multiple generations throughout a season and will therefore be present for a longer period. Including these random effects allows different families to vary either in the intercept or the slopes. We compared five models for each predictor where we varied whether the family random effect was: (a) only for the intercepts (1|family), (b) a random slope for the number of days but no covariance in between the intercept and slope (0 + days|family), (c) a random slope for the first Julian day with no covariance between the intercept and slope (0 + first Julian|family), (d) a random slope for days with covariance between the intercept and slope (1 + days|family), and (e) a random slope for first Julian day with covariance between the intercept and slope (1|first Julian|family). For *c* and *d*′, variables that are proportions (values between 0 and 1), we used nonlinear mixed effects models, with the same formula as above, but specifying a binomial distribution. Since the proportions in these variables are not derived from counts, a more appropriate distribution is the beta distribution. We tried to fit these classes of models using a beta distribution; however, due to poor convergence we could not fit all of the models presented above. We fit the simplest random effect structure, and the coefficients are similar in magnitude and direction as those obtained from the binomial distribution. Therefore, we present the results obtained using the binomial distribution. We selected the best fitting model for the family random effect using AIC (Aho et al., 2014) (Table S2). Models were run separately for plants and pollinators, and for each network metric separately, for a total of eight models. The number of days and first Julian day were log_10_ transformed to improve assumptions of normality and homoscedasticity of residuals.

#### 2.5.2 Robustness analysis

To measure network robustness (Memmott et al., 2004), we used the “Dependent random‐search Coextinction Model” (Baumgartner et al., 2020). This algorithm randomly removes either a pollinator or a plant *j* and then estimates the susceptibility to extinction of the interacting species *i*. The susceptibility to extinction of species *i* is determined by the product of the interaction strength, the intrinsic demographic dependence of the affected species (Ri ) on the interaction with the removed species, and the average dissimilarity between the removed species *j* and all other species. Demographic dependence (Ri) determines how strongly a plant or pollinator species depend on their interacting species for their growth. For example, if a plant is able to produce viable seeds through selfing or apomixis, then its demographic dependence will be lower. Based on the species *i* susceptibility to extinction, a Bernoulli trial will decide whether the species *i* indeed goes extinct. If the species *i* is determined to go extinct based on the Bernoulli trial, the algorithm will then deal with this situation in two ways. First, if the species *i* that is susceptible to extinction only interacted with the removed species *j*, the species *i* that is susceptible to extinction can only persist if it creates new interactions. The algorithm only allows species that have a susceptibility less than the extinction threshold to create new interactions. The creation of new interactions depends on the similarity between the removed species j and the others in the network; in this analysis, it included similarity of the interaction matrix or overlapping phenology (see description below). The maximum number of new interactions is constrained to be the original degree of the species *i*(*n*) plus one (*n* + 1). Second, if the species *i* that is susceptible to extinction also interacted with other species, then the algorithm will enhance the interaction strength with these other species only if the susceptible species is allowed to persist following a Bernoulli trial.

The algorithm then uses the following parameters: the extinction threshold, the intrinsic demographic dependence (Ri), the interaction matrix, and the dissimilarity between species of the same type (plants or pollinators). Following Baumgartner et al. (2020), we used three levels extinction threshold 0.25, 0.5, and 0.75, and we used three levels of intrinsic demographic dependence (Ri), which were randomly sampled from a uniform distribution, low (0 < Ri < 0.3), intermediate (0.3 < Ri < 0.6), and high (0.6 < Ri < 0.9). We used every combination of these two sets of parameters resulting in nine parameter combinations. For the dissimilarity between the species, we used two options: first, the default that calculates the distance between the species based on the interaction matrix (species that interact with the same suite of species will be more similar, and this is a pairwise Bray–Curtis dissimilarities among species within either pollinators or plants), and second, we used the dissimilarity between species based on phenological variables (days and First Julian dates). We ran the algorithm for the two types of distances using all nine parameter combinations (resulting in 18 combinations).

As mentioned above, the algorithm randomly removes species at the beginning of the run in order to cause the extinction cascade. In order to test the effect of removing species based on phenological order (i.e., removing species based on the number of days or First Julian order), we modified the algorithm so that the original extinction was not random but instead proportional to these phenological variables. We were interested in four scenarios with respect to network robustness: (a) species are removed from a network according to when they are first active—that is, the species that were first active during the **earliest date** of the season have a higher probability of being removed first (earliest date first); (b) species are removed from a network according to the reverse order of activity—that is, the species that were first active on the **latest date** have a higher probability of being removed first (latest date first); (c) species are removed from a network according to total duration of activity during the season—that is, the species active the **least** number of days have a higher probability of being removed first (shortest duration first); and (d) species are removed from a network according to reversed order of total duration of activity during the season—that is, the species active the **most** number of days have a higher probability of being removed first (longest duration first). In total, we ran 90 different parameter combinations for 33 networks.

To calculate the probability of secondary extinction, the algorithm was run 1,000 times, and we counted the number of times that a secondary extinction occurred. To differentiate between the secondary extinctions caused by the removal of a pollinator or a plant, we divided the algorithm runs on whether the original extinction was that of a pollinator or a plant. Since the algorithm does not draw the original extinction based on total species richness, but instead it first does a binomial draw between plant and pollinator, and the number of original extinctions of plants and pollinators was roughly equal.

## 3 RESULTS

The networks in oak savanna had on average 19.8 plant species (2.96 sd) and 61.8 pollinators species (13.8 sd), in shrub–steppe 22 plant species (6.09 sd) and 83 pollinator species (25.2 sd), and in hedgerow sites 13.8 plant species (6.18 sd) and 22 pollinator species (9.54 sd). All networks except one in a hedgerow site had more pollinator species than plant species.

### 3.1 Network structure and phenology analysis

**FIGURE 3:**
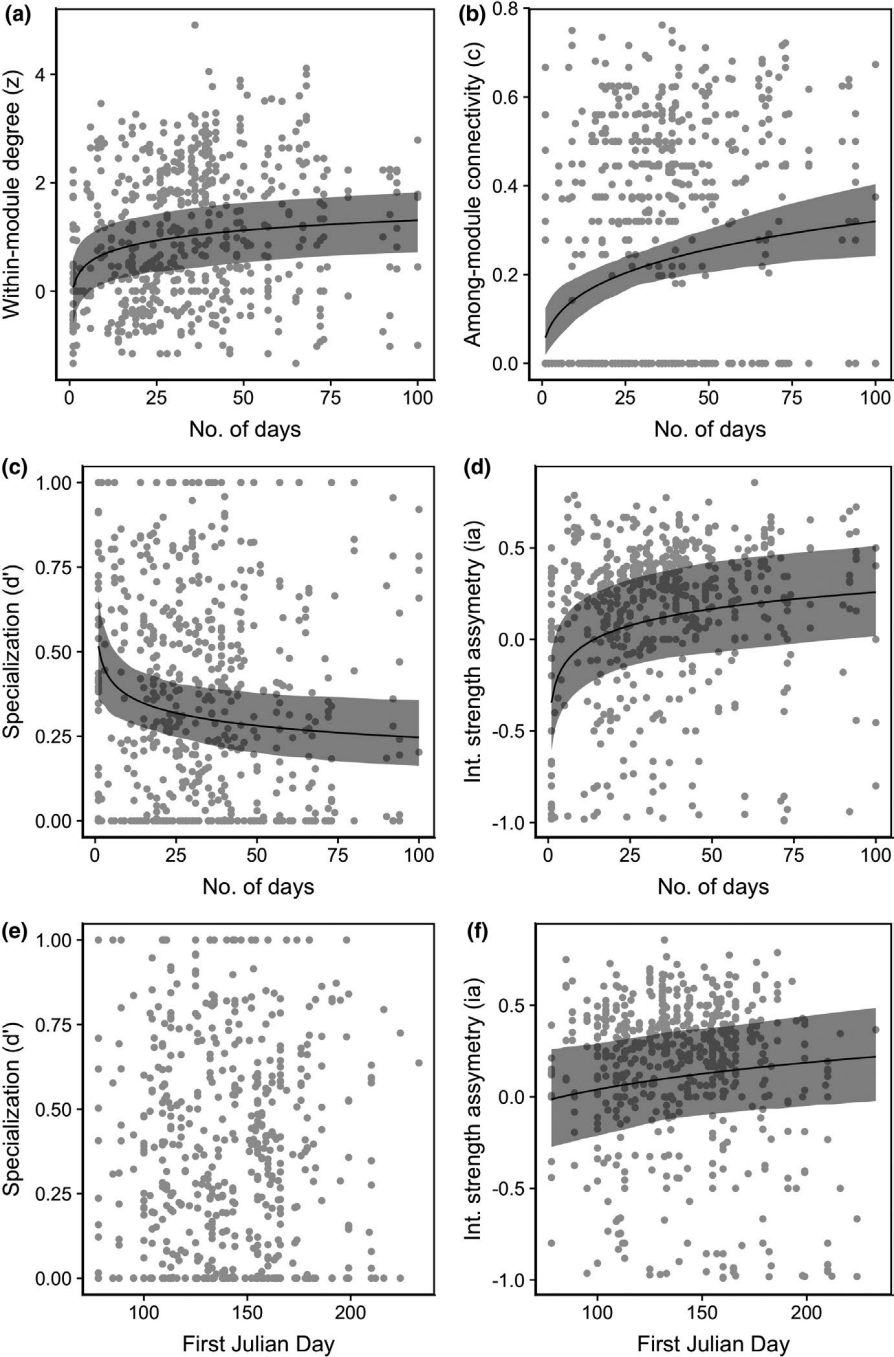
Various network metrics in relation to pollinator and plant phenological traits for plants. (a–d) Number of days observed in a network vs. (a) within‐module degree (*z*), (b) among‐module connectivity (*c*), (c) specialization (*d*′), and (d) interaction strength asymmetry (ia). (e–f) The date of first appearance in a network vs. (e) specialization (*d*′), and (f) interaction strength asymmetry (ia). Shaded areas represent bootstrapped confidence intervals at 95%

We ranked models based on AICc, and identified top models based on a criteria of ΔAICc < 2.0 from the best model (Burnham & Anderson, 2002) (Table S2). For plants, the best model for *z* was 1 + log10(first_Julian)|FamilyName, for c and *d*′ was 0 + log10(days)|FamilyName, and for ai was 1 + log10(days)|FamilyName. Therefore, the slopes for *z* with first Julian varied between families, while for the other metrics, the slopes with the duration activity varied between families. For pollinators, the best model for *z*, *d*′, and ia was 0 + log10(days)|FamilyName and for *c* was 1 + log10(days)|FamilyName. Therefore, for pollinators all of the slopes varied within families, but for *c*, the intercepts and the slopes were correlated while for the other metrics, it was not. This result highlights that the relationship between species roles in a network and their phenology varies between families. On average, species‐level network metrics were positively related to phenology variables. For both plants and pollinators, within‐module degree (*z*) and among‐module connectivity (*c*) were positively related to the number of days in a network (plant and pollinator *p* < 0.001), such that plants and pollinators that were active longer in the season were more connected within the module and among modules (Table 1; Figures 2, 3).

**TABLE 1:** Results of regression analyses of the relationship between two phenology variables (first day of appearance in a network and total number of days observed in a network) and four species‐level properties (specialization (*d*′), among‐module connectivity (*c*), within‐module degree (*z*), and interaction asymmetry (ia))

Variable	*d*′		*c*		*z*			ia		
	Est.	*P*	Est.	*P*	Est.	*df*	*P*	Est.	*df*	*p*
Plants										
First Julian	1.265	.312	1.824	.099	0.007	36.075	.994	0.487	304.856	.019*
Days	−0.591	.008**	1.014	<.001***	0.622	569.192	<.001***	0.302	21.181	<.001***
Pollinators										
First Julian	−1.998	.035*	−1.493	.239	−0.200	1,569.627	.174	−0.552	1,450.512	<.001***
Days	−0.396	.001***	2.343	<.001***	0.135	148.003	<.001***	0.162	107.821	<.001***

Note

This table represents eight separate statistical models, one for each of pollinators and plants, and one for each response variable. *p < 0.05; **p < 0.01; ***p < 0.001.

**FIGURE 4:**
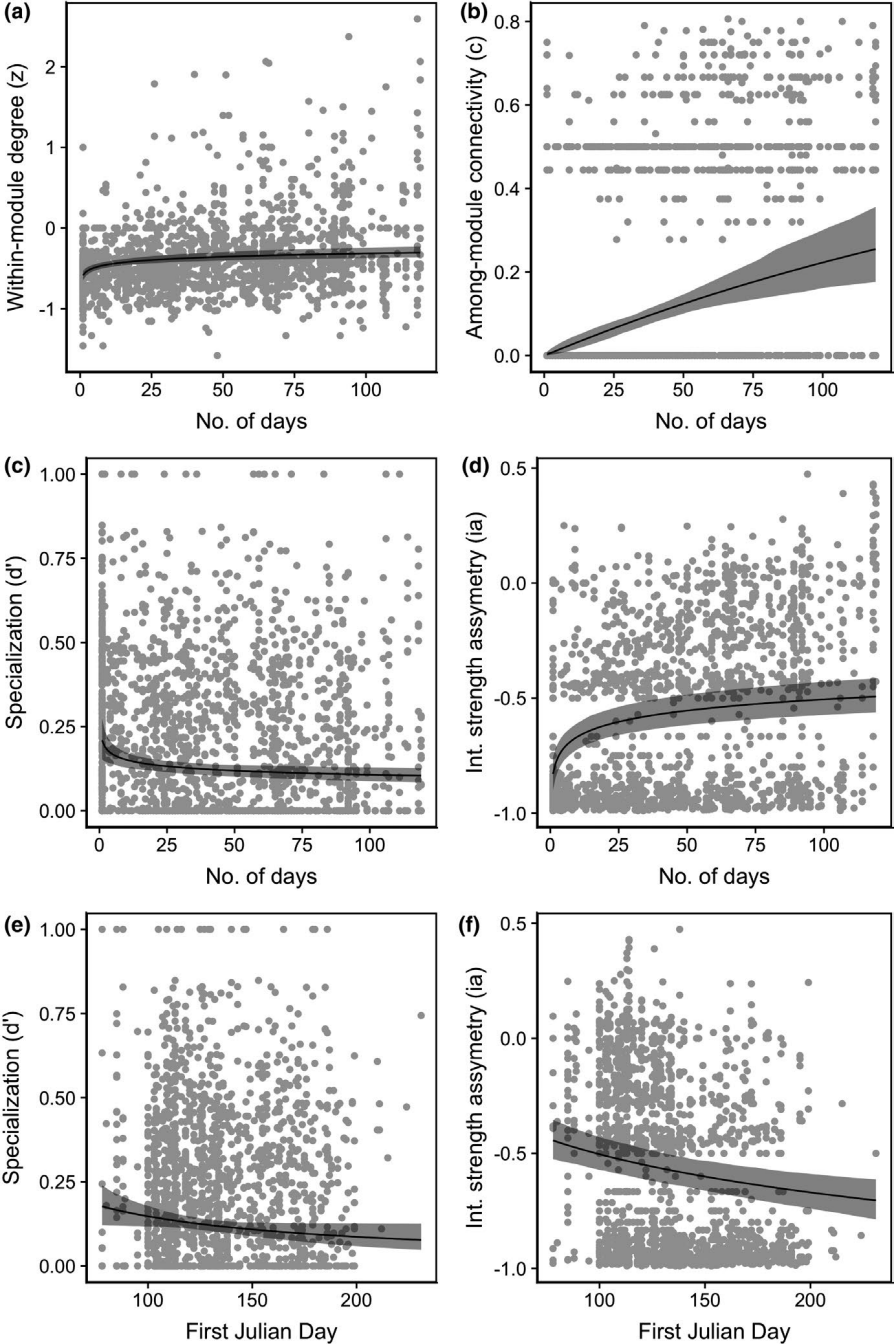
Various network metrics in relation to pollinator and plant phenological traits for pollinators. (a–d) Number of days observed in a network vs. (a) within‐module degree (*z*), (b) among‐module connectivity (*c*), (c) specialization (*d*′), and (d) interaction strength asymmetry (ia). (e–f) The date of first appearance in a network vs. (e) specialization (*d*′), and (f) interaction strength asymmetry (ia). Shaded areas represent bootstrapped confidence intervals at 95%

The degree of specialization (*d*′) for plants and pollinators was negatively related to the number of days in a network (*p* = 0.008, *p* = 0.001 respectively), such that plants and pollinators that were active longer in the season were more generalized. For pollinators, the degree of specialization (*d*′) was negatively related to the day of first appearance (*p* = 0.035), such that pollinators that appeared later in the season were more generalized (Table 1; Figures 2, 3, 4).

Last, interaction asymmetry was positively related to the number of days active for both pollinators and plants (both *p* < 0.001), such that plants and pollinators that were active longer in the season had a greater effect on the species they interacted with, compared with the effect those species had on them. The date of first appearance was positively related to interaction asymmetry for plants (*p* = 0.019) and negatively related to pollinators (*p* < 0.001). Plants that appeared later in the season had a stronger effect on pollinators they interact with, while pollinators that appeared early in the season had a stronger effect on the plants they interact with. On average, pollinators were more strongly impacted by the plants they interacted with (Figure 3d,f, points below zero) and plants had a stronger effect on pollinators (Figure 4d,f, points above zero).

### 3.2 Robustness analysis

**FIGURE 5:**
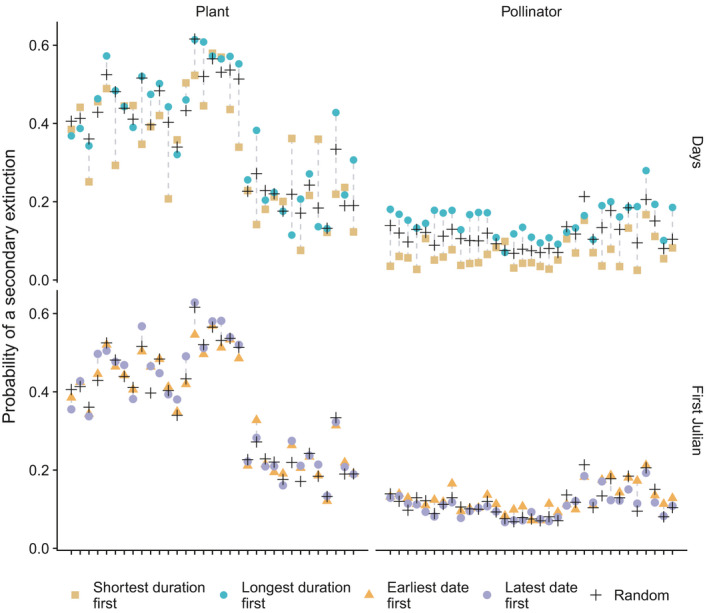
Probability of secondary extinction of 33 plant–pollinator networks in response to removal of species according to either first Julian date of appearance in a network (earliest date first, orange triangles), last Julian date of appearance (latest date first, purple circles), least to most days observed in network (shortest duration first, gold squares; last day minus first, in number of days), most to least days observed in network (longest duration first, green circles; last day minus first, in number of days), and at random (random, black crosses). Note: Networks in each of the four panels are in the same order as Figure 1; scales in all four panels are the same; drop lines connect points that belong to the same network in each panel. In this analysis, the extinction threshold was 0.75, Ri was 0.6–0.9, and the distance between species was based on the interaction matrix. For all other parameter combinations, see supplementary material

Plant removal resulted in a higher probability of extinction than pollinator removal, suggesting that networks are more robust to removal of pollinators than to removal of plants. This result is probably due to the differences in species richness between the two groups, that is, there are more pollinators than plant species in the networks. Interestingly, when plants and pollinators were removed based on the number of days they appeared in the network, more networks (30–33 pollinator networks and 21–27 plant networks out of 33 total networks) had a higher probability of secondary extinction when *either* plants and pollinators were removed from the most number of days to the least number present in the network, compared with removal from the least number of days to the most (Figure 5, Table S3). On the other hand, when plants and pollinators were removed based on their First Julian date, we found that more networks had a higher probability of secondary extinction when pollinators were removed starting from the beginning of the season, while more networks had a higher probability of secondary extinction when plants were removed starting from the end of the season (Figure 5, Table S3).

Allowing new interactions to occur based on how different species are in their phenology reduced the probability of secondary extinctions compared with allowing new interactions based on the current interaction matrix (Figure S2).

The probability of secondary extinction depended on the extinction threshold and the demographic dependence between the species (Ri). This result confirms the model is working as expected. We found that as the extinction threshold increased, the probability of secondary extinctions decreased (Figure S2) and, as Ri increased, the probability of secondary extinction increased (Figure S2). However, the main results were largely consistent regardless of the combination of extinction threshold and Ri.

## 4 DISCUSSION

### 4.1 Network structure and phenology analysis

Phenology was an important predictor of species‐level network properties for both plants and pollinators in this study. This is consistent with findings from simulation studies (Encinas‐Viso et al., 2012) that found that phenology could be extremely important in structuring networks. For plants, we found that species active longer were more connected both within their module and among modules. Plant species that are active longer are important structurally in the network as they interact with multiple species, exert strong effects on interaction partners, and are generalists (González et al., 2010). Some of the species that were active the longest were *Cornus stolonifera* (Family: Cornaceae), *Symphoricarpus albus* (Caprifoliaceae), and *Ranunculus repens* (Ranunculaceae). *Ranunculus* is a weedy forb while *Symphoricarpus* and *Cornus* are long‐lasting shrubs. These species additionally have radially symmetrical flowers allowing many types of pollinators to access the flowers (Lovett‐Doust et al., 1990; Sargent, 2004; Stewart‐Wade et al., 2002; Wolfe & Krstolic, 1999). For pollinators, species that were active longer in the season also had more connections among and within modules, and were less specialized. Some of the species that were active the longest were *Bombus centralis* (Apidae), *Sphaerophoria weemsi* (Syrphidae), and *Lasioglossum pacatum* (Halictidae). Bumble bees (*Bombus*) tend to be highly generalized in floral visit patterns (Laverty & Plowright, 1988), have multiple generations of workers per season (Michener, 2000), and although individual workers may specialize on particular floral resources, the species as a whole uses diverse plants over an extended flight period. Hover flies (Syrphidae) are multivoltine and use a diverse array of generalized flowers. In contrast, solitary bees in our region are active for only a few weeks, making it unsurprising that they are not within‐ or among‐module connectors. Similarly, we found that pollinators were less specialized (more generalized) when they first appeared later in the season which in our data would include wasps and some hover flies. Most of our results point to the relationship between phenological traits and the role of species in a network being mediated by taxonomy.

Plants and pollinators had opposite relationships between date of emergence (Julian day) and interaction strength asymmetry. Plant species that emerged later in the season (i.e., their first Julian day was higher) had a higher interaction strength asymmetry. That is, species that emerged later in the season affected their interacting partners more strongly than their partners affected them. Some of the plant species that emerged later in the season were *Galeopsis tetrahit* (Lamiaceae), *Lythrum salicaria* (Lythraceae), and *Polygonum persicaria* (Polygonaceae); these are all non‐native herbaceous species with multi‐flower inflorescences that may provide a large resource pulse. In contrast, we found that for pollinators, the interaction strength asymmetry decreased as species emerged later (i.e., their first Julian day was higher). Some of the pollinator species that emerged were solitary mining bees, such as *Andrena nigrihirta, Andrena merriami, Andrena sladeni, Andrena trizonata*, and *Andrena porterae* (Andrenidae). In two of the ecosystems studied, oak savanna and shrub–steppe, we observed that at the beginning of the season, many plant species bloom in high density, but temperatures are not yet reliably warm enough for consistent insect activity. This may lead to plant reproduction being pollinator limited (Kudo & Ida, 2013; Schemske et al., 1978). By the end of the season, the amount of food and nutrients available for the pollinators is lower (floral density generally declines in these ecosystems, e.g., Wray & Elle, 2015), but some pollinator populations have grown over time (e.g., social bees and wasps). It may be that late‐emerging plant species provide important resources for pollinators (Garbuzov & Ratnieks, 2014; Mattila & Otis, 2007). We hypothesize that changes in resources available over time, and the difference in what those resources are (food for pollinators, but mating opportunities for plants), are an important driver of robustness in networks; this should be studied.

### 4.2 Robustness analysis

We observed that networks were on average more robust to removal of pollinators than to removal of plants. This makes sense because plants more often link together the plant–pollinator network (hubs organize around plants), whereas pollinators are less often important hubs (see Figure 2). This pattern was also seen in another study—Olesen et al. (2007) found that plant species were more often module hubs and network hubs than pollinators (see Figure 2 in Olesen et al., 2007). We found all networks (except one hedgerow) had more pollinator species than plant species, and in turn, plants on average had a higher degree than pollinators (Table S1). This result is consistent with other studies in which networks had more pollinator species than plant species (Basilio et al., 2006). Because of this asymmetry in the number of plants versus pollinators, we would expect that individual plant species would play a more important role than individual pollinator species (Vázquez & Aizen, 2004).

Removing both plants and pollinators in order from the least to most days observed in the network (shortest duration first) resulted in more robust networks than removing plants and pollinators in order from the most to least days observed (longest duration first). This is consistent with our previous results, which found that plants and pollinators that are active the most number of days were more connected both within and among modules, were less specialized, and were affected more strongly by interacting partners. Therefore, removing plants and pollinators that are well connected (i.e., are present the most number of days) results in less robust networks. While a phenological change is distinct from a complete removal, a species phenological change can result in species flowering or emerging earlier, but also have a shorter duration of their activity (e.g., Burkle et al., 2013). Both the shift, but also the reduction in activity, have been shown to reduce the robustness of networks (Revilla et al., 2015).

Removing plants according to the first Julian date of appearance in a network (earliest date first) resulted in *more* robust networks than removing plants in order from the last Julian date of appearance (late date first). Plants that appeared later in the season were more important for network robustness than the plants that appeared earlier in the season. On the other hand, removing pollinators according to the first Julian date of appearance in a network (earliest date first) resulted in *less* robust networks than removing plants in order from the last Julian date of appearance (late date first). These results are consistent with our results for plants and pollinators relating interaction strength asymmetry to the first Julian day of appearance. The mechanism is hypothesized to be the same that different resources are limiting for plants versus pollinators at different times over the flowering/flight season (see above). Our results are consistent with Ramos‐Jiliberto et al. (2018), who found that plant persistence was most sensitive to the disappearance of pollinators that start earlier or finish later in the season, and that pollinators were most sensitive to the disappearance of plants that started early and had long seasons of bloom.

Allowing networks to produce new interactions based on phenology increased the robustness of the networks, comparing to the robustness when new interactions were produced solely based on the pattern of current interactions, consistent with what Vizentin‐Bugoni et al. (2020) found. This result, coupled with the fact that phenological constraints alone can produce realistic networks (Olito & Fox, 2015), suggests that networks might be more robust to extinctions than previously thought if species can shift their interaction partners.

Plant–pollinator networks are at risk both due to the global decline of pollinators and phenological shifts due to climate change. Some pollinator species including some bumble bees are declining (*Bombus* spp.; Williams & Osborne, 2009, Arbetman et al., 2017). The loss of bumble bees worldwide can have cascading consequences on plant–pollinator networks since bumble bees are active for long periods, which we found to be a trait of module connectors in our networks. From our analysis, we found that removing species with long activity periods reduced the robustness of the networks; therefore, losing connectors like bumble bees can reduce the robustness of networks. In addition, climate change impacts the phenology of many plant species, affecting in particular spring events such as flowering time (Gordo & Sanz, 2010). Phenological shifts can result in earlier flowing and emergence time for plants and pollinators, but it can also result in decreases in the duration of their activity (Burkle et al., 2013). Earlier flowering time can increase the temporal mismatch between plants and pollinators (Bartomeus et al., 2011; Kudo & Ida, 2013). Our networks have a higher probability of extinction when pollinators are removed early in the season, and other research indicates that plants are typically pollen limited in the spring (Schemske et al., 1978). This suggests that shifts to earlier flowering time could have higher consequences for plant fitness than shifts in pollinator emergence to earlier in the season. In the Garry Oak ecosystems for example, *Collinsia parviflora* has shifted its phenology to earlier in the season in dry sites that truncate the length of the flowering season (Elle, 2004). Perhaps to mitigate the potential for pollen limitation, there have been correlated shifts to early autonomous selfing (no longer requiring pollinators) in these dry sites. This highlights the need to estimate the demographic dependence between plants and pollinators, as phenological mismatches assume that the fitness of the plant depends on the pollinator (or vice versa) for the community as a whole (Kharouba & Wolkovich, 2020). Estimating the plant fitness that depends on the pollinator would not only allow us to determine whether phenological mismatches are likely to occur, but it would also provide more realistic predictors of the probability of secondary extinctions (Baumgartner et al., 2020).

### 4.3 Caveats

One main caveat of these results is the confounding effect of abundance, as abundance is known to determine metrics of network structure (Olito & Fox, 2015), as well as species degree (Bascompte et al., 2003; Jordano et al., 2003; Vázquez & Aizen, 2003; Vázquez & Aizen, 2004). However, some of our metrics do include the frequency of the interactions, such as the standardized specialization for each species (*d*′). Another caveat of these results is that we used the first and last day as estimates of phenology. These types of estimates can be biased by outliers (Pearse et al., 2017; Taylor, 2019). However, using other methods such as the Weibull distribution requires multiple observations, which we did not have for all species. While our estimates of phenology can be biased, they are consistent across all species and guilds. Finally, we want to add that sampling effort can bias network metric calculations (Falcão et al., 2016; Vizentin‐Bugoni et al., 2016), but networks that are better sampled are more robust to these biases. Here, we use webs that were sampled nine to twelve times in a year, with effort standardized within a study. We also used quantitative metrics that are more robust than binary metrics (Vizentin‐Bugoni et al., 2016). In order to be able to compare the results from the different ecosystems, we divided the frequency of the interactions by the number of samples and we also used study as a random effect. By accounting for the variation produced through sampling and study, we improve the generality of our conclusions.

## 5 CONCLUSION

Our results show that across a large sample of 33 networks, species phenology can be an important predictor of network structure. In particular, the number of days a species is active in a network predicted how connected the species is, for both plants and pollinators. We also found that plant impacts are larger at the end of the season, while pollinator impacts are important early in the season. Our study suggests that if the duration of species activity is reduced or shifted due to climate change, we will see large subsequent effects on network metrics associated with robustness. Future work should build on our findings by exploring how experimental or natural changes in phenological variables, like time of first appearance or duration of activity in a community, influence network structure. Specifically, experiments can remove early flowering plants or late‐flowering plants and assess whether pollinators form new interactions with plants that share phenology.

## CONFLICT OF INTEREST

The authors declare no conflict of interest.

## AUTHOR CONTRIBUTIONS

**Laura Melissa Guzman:** Formal analysis (equal); Methodology (equal); Software (equal); Visualization (equal); Writing‐original draft (equal); Writing‐review & editing (equal). **Scott A. Chamberlain:** Conceptualization (equal); Formal analysis (equal); Methodology (equal); Software (equal); Visualization (equal); Writing‐original draft (equal). **Elizabeth Elle:** Conceptualization (equal); Data curation (equal); Funding acquisition (equal); Methodology (equal); Project administration (equal); Resources (equal); Writing‐original draft (equal); Writing‐review & editing (equal).

## Supporting information

Supplementary Material

## Data Availability

Interaction data are available at https://doi.org/10.5061/dryad.c59zw3r7t.
