# Supplementary material for: Network robustness and structure depend on the phenological characteristics of plants and pollinators
Source: Ecol Evol. 2021 Sep 10;11(19):13321–34. doi: 10.1002/ece3.8055 (PMC8495816; doi:10.1002/ece3.8055)

Supplementary material for Network robustness and structure depends on the phenological characteristics of plants and pollinators.

Figure S1 Map of the study sites. The entire map of the province of British Columbia (A) with the subsection zoomed in B. Sites collected in 2009/2010 (purple) were collected in the oak savannah, sites collected in 2010 (green) were collected in antelope brush shrub steppe and sagebrush steppe, and sites collected in 2013 (yellow) were collected in hedgerow restorations. Mapping shape files were obtained from iMapBC (https://www2.gov.bc.ca/gov/content/data/geographic-data-services/web-based-mapping/imapbc).

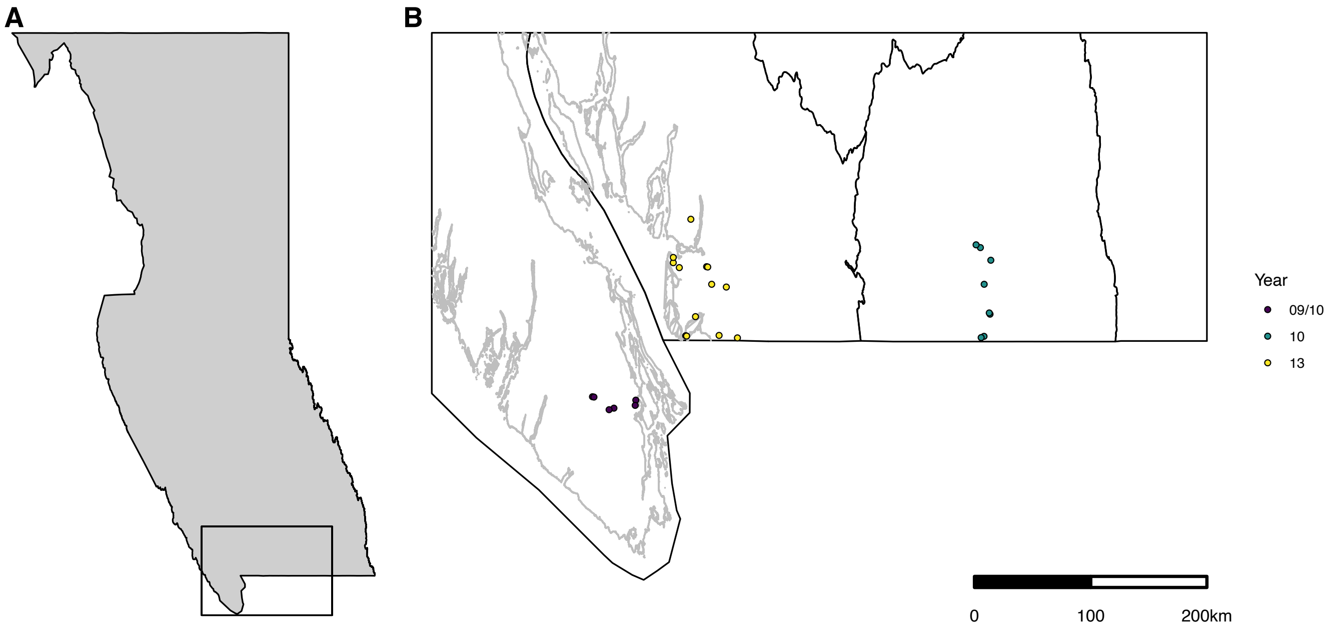

Table S1 Details of each of the study sites in British Columbia, Canada. Abbrevations: oak sav. = oak savannah; antel. steppe = antelope brush shrub steppe; sage. steppe = sagebrush

steppe, hedg res = hedgerow restorations, as well details on the networks such as modularity and modularity significance, the number of species for each plant and pollinators and average degree for each network for the 33 networks

Ecosystem	Year(s) Coll.	Modularity	Significance	Number of plants	Mean degree plants	Number of pollinators	Mean degree pollinators
oak sav.	09/10	0.525	2.88e-11	46	6.118	17	2.261
oak sav.	09/10	0.626	1.68e-02	50	5.300	20	2.120
oak sav.	09/10	0.488	1.03e-21	50	4.941	17	1.920
oak sav.	09/10	0.601	4.61e-08	66	5.500	20	1.924
oak sav.	09/10	0.574	4.22e-18	75	6.783	23	2.147
oak sav.	09/10	0.533	5.19e-20	81	7.708	24	2.321
oak sav.	09/10	0.689	1.18e-01	45	4.214	14	1.578
oak sav.	09/10	0.670	2.19e-09	44	3.706	17	1.705
oak sav.	09/10	0.623	3.98e-02	72	7.050	20	2.208
oak sav.	09/10	0.561	2.68e-13	65	5.591	22	2.046
oak sav.	09/10	0.523	1.93e-25	77	7.636	22	2.416
oak sav.	09/10	0.554	1.57e-25	70	5.238	21	1.871
antel. steppe	10	0.704	1.48e-01	34	3.727	11	1.206
antel. steppe	10	0.582	7.18e-01	56	6.214	14	1.554
antel. steppe	10	0.662	3.09e-03	98	7.583	24	1.837
antel. steppe	10	0.620	1.27e-27	88	6.615	26	1.955
sage. steppe	10	0.597	2.09e-13	97	7.560	25	1.907

sage. steppe	10	0.548	3.18e-04	107	7.679	28	1.963
sage. steppe	10	0.568	1.29e-34	85	8.391	23	2.247
sage. steppe	10	0.591	6.52e-19	100	7.680	25	1.870
hedg. res.	13	0.497	2.68e-12	26	3.846	13	1.577
hedg. res.	13	0.494	5.82e-26	44	4.520	25	2.205
hedg. res.	13	0.502	4.35e-04	15	2.917	12	2.000
hedg. res.	13	0.483	9.41e-02	14	3.833	6	1.429
hedg. res.	13	0.404	9.28e-10	10	2.556	9	1.800
hedg. res.	13	0.543	8.83e-01	16	2.909	11	1.812
hedg. res.	13	0.360	2.43e-14	20	4.000	11	2.050
hedg. res.	13	0.499	7.16e-06	18	3.308	13	1.833
hedg. res.	13	0.589	1.52e-01	19	4.273	11	2.158
hedg. res.	13	0.522	8.78e-08	18	2.500	24	2.556
hedg. res.	13	0.437	2.78e-23	35	4.944	18	2.114
hedg. res.	13	0.488	3.49e-02	20	4.833	6	1.300
hedg. res.	13	0.470	2.42e-05	31	4.700	20	2.548

Table S2

We compared five models for each predictor where we varied whether the family random effect was: (i) only for the intercepts (1|family), (ii) a random slope for the number of days but no covariance in between the intercept and slope (0+days|family), (iii) a random slope for the first Julian day with no covariance between the intercept and slope (0+first Julian | family), (iv) a random slope for days with covariance between the intercept and slope (1 +

days | family) and (v) a random slope for first Julian day with covariance between the intercept and slope (1 | first Julian |family). We selected the best fitting model for the family random effect using AIC

Model structure was: response ~ log10(first_Julian) + log10(days) + (1 | pi/.id) + Family random effect.

Plants

Model	Family random effect	z		c		d’		ia	
		df	AIC	df	AIC	df	AIC	df	AIC
1	(1 | FamilyName)	7	1848.628	6	606.699	6	750.7722	7	517.8107
2	(0 + log10(days) | FamilyName)	7	1848.566	**6**	**606.699**	**6**	**748.7950**	7	530.1231
3	(0 + log10(first_Julian) |FamilyName)	7	1849.441	6	606.699	6	751.2407	7	517.7547
4	(1 + log10(days) | FamilyName)	9	1847.378	8	610.699	8	752.1878	**9**	**505.4113**
5	(1 + log10(first_Julian) | FamilyName)	**9**	**1845.088**	8	610.699	8	755.2375	9	520.9011

Pollinators

Model	Family random effect	z		c		d’		ia	
		df	AIC	df	AIC	df	AIC	df	AIC
1	(1 | FamilyName)	7	2027.596	6	1099.950	6	1332.472	7	1105.336
2	(0 + log10(days) | FamilyName)	**7**	**1996.510**	6	1099.950	**6**	**1332.472**	**7**	**1072.677**
3	(0 + log10(first_Julian) |FamilyName)	7	2030.213	6	1099.950	6	1332.472	7	1108.556
4	(1 + log10(days) | FamilyName)	9	1998.208	**8**	**1089.255**	8	1336.472	9	1076.677

5	(1 + log10(first_Julian) | FamilyName)	9	2017.617	8	1103.950	8	1336.472	9	1090.839

Table S3 The number of networks (out of 33) where the probability of secondary extinction is higher when species are removed from 'low to high' number of days of First Julian date or the opposite, where the number of networks where the probability of secondary extinction is higher when species are removed from 'high to low', across all of the parameter combinations.

Days:

Short > Long = removing species where short duration first results in a higher probability of extinction than removing species by the longest duration first.

			Distance is interactions		Distance is phenology	
**Extinction threshold**	**Ri**	**Guild of first removal**	**Short > Long**	**Long > Short**	**Short > Long**	**Long > Short**
0.25	0 -0.3	Pollinator	2	31	6	27
0.25	0 -0.3	Plant	6	27	12	21
0.50	0 -0.3	Pollinator	3	30	7	26
0.50	0 -0.3	Plant	9	24	14	19
0.75	0 -0.3	Pollinator	1	32	8	25
0.75	0 -0.3	Plant	9	24	9	24
0.25	0.3-0.6	Pollinator	0	33	2	31
0.25	0.3-0.6	Plant	8	25	13	20
0.50	0.3-0.6	Pollinator	0	33	3	30
0.50	0.3-0.6	Plant	10	23	12	21
0.75	0.3-0.6	Pollinator	1	32	6	27
0.75	0.3-0.6	Plant	8	25	13	20

0.25	0.6-0.9	Pollinator	1	32	5	28
0.25	0.6-0.9	Plant	11	22	13	20
0.50	0.6-0.9	Pollinator	3	30	3	30
0.50	0.6-0.9	Plant	11	22	8	25
0.75	0.6-0.9	Pollinator	1	32	3	30
0.75	0.6-0.9	Plant	12	21	9	24

First Julian:
Early > Late = removing species where that appear earlier in the season first results in a higher probability of extinction than removing that appear late in the season first.

			**Distance is interactions**		**Distance is phenology**	
**Extinction threshold**	**Ri**	**Guild of first removal**	**Early > Late**	**Late > Early**	**Early > Late**	**Late > Early**
0.25	0 -0.3	Pollinator	22	11	18	15
0.25	0 -0.3	Plant	14	19	17	16
0.50	0 -0.3	Pollinator	25	8	21	12
0.50	0 -0.3	Plant	15	18	14	19
0.75	0 -0.3	Pollinator	17	16	21	12
0.75	0 -0.3	Plant	14	19	25	8
0.25	0.3-0.6	Pollinator	24	9	17	16
0.25	0.3-0.6	Plant	12	21	10	23

0.50	0.3-0.6	Pollinator	26	7	19	14
0.50	0.3-0.6	Plant	12	21	14	19
0.75	0.3-0.6	Pollinator	20	13	24	9
0.75	0.3-0.6	Plant	13	20	16	17
0.25	0.6-0.9	Pollinator	22	11	25	8
0.25	0.6-0.9	Plant	16	17	14	19
0.50	0.6-0.9	Pollinator	27	6	18	15
0.50	0.6-0.9	Plant	11	22	12	21
0.75	0.6-0.9	Pollinator	28	5	20	13
0.75	0.6-0.9	Plant	11	22	14	19

Figure S2 Probability of secondary extinction of 33 plant-pollinator networks in response to removal of species according to either first Julian date of appearance in a network (earliest date first, orange triangles), last Julian date of appearance (latest date first, purple circles), least to most days observed in network (shortest duration first, gold squares; last day minus first, in number of days), most to least days observed in network (longest duration first, green circles; last day minus first, in number of days), and at random (random, black crosses). The figures A-R use different model parameters than Figure 5.

A) Extinction threshold = 0.25, Ri = 0-0.3, distance based on the interaction matrix

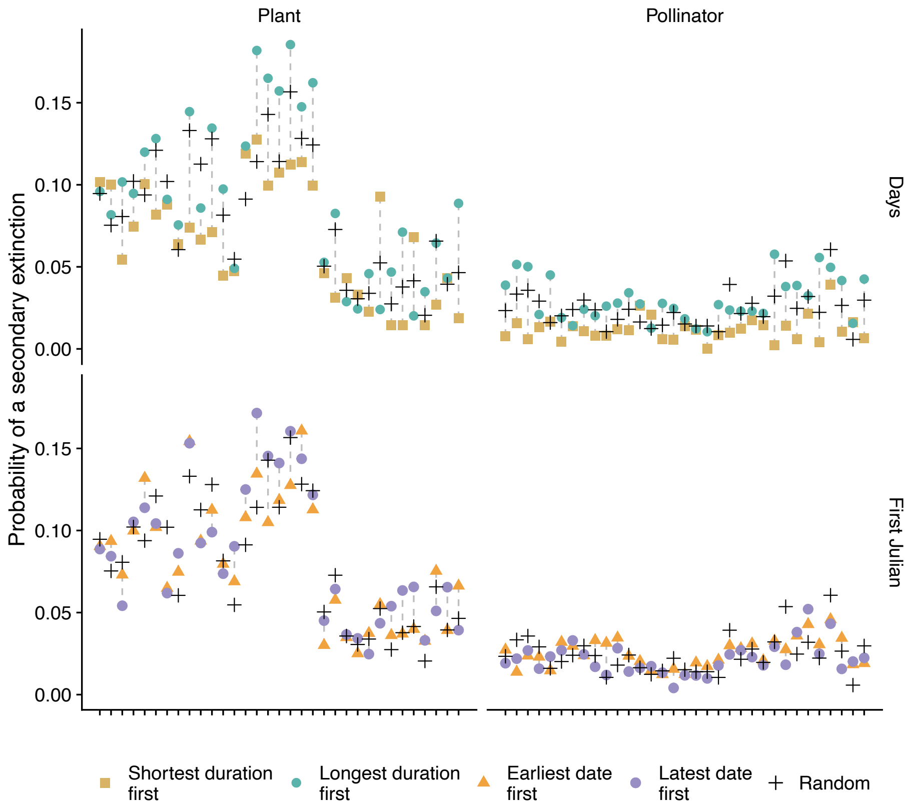

B) Extinction threshold = 0.5, Ri = 0- 0.3, distance based on the interaction matrix

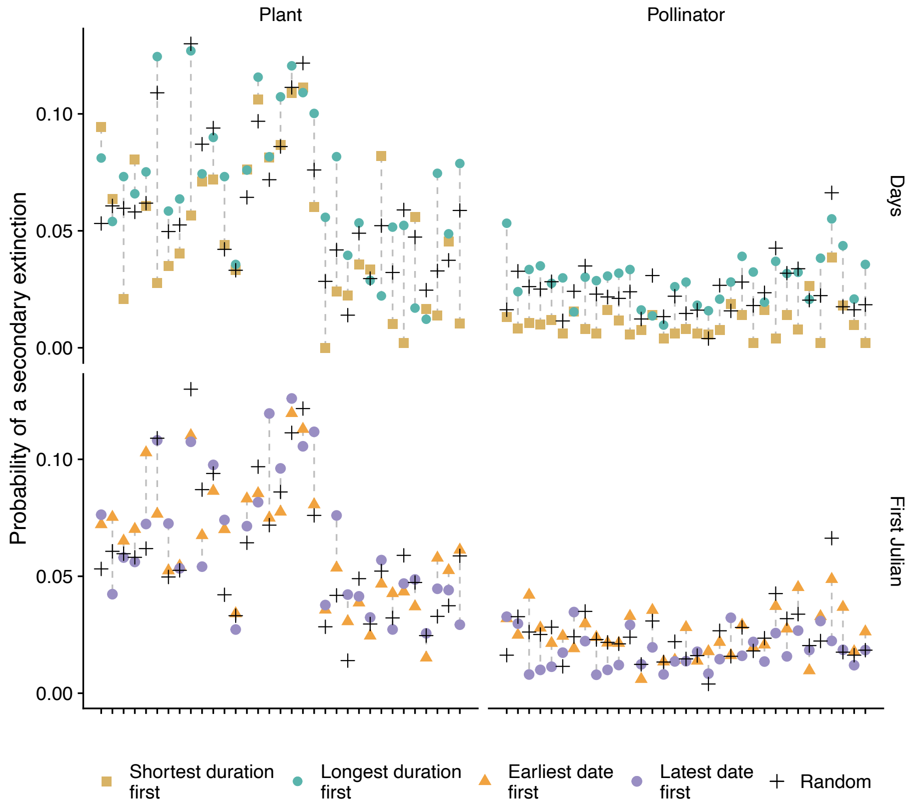

C) Extinction threshold = 0.75, Ri = 0- 0.3, distance based on the interaction matrix

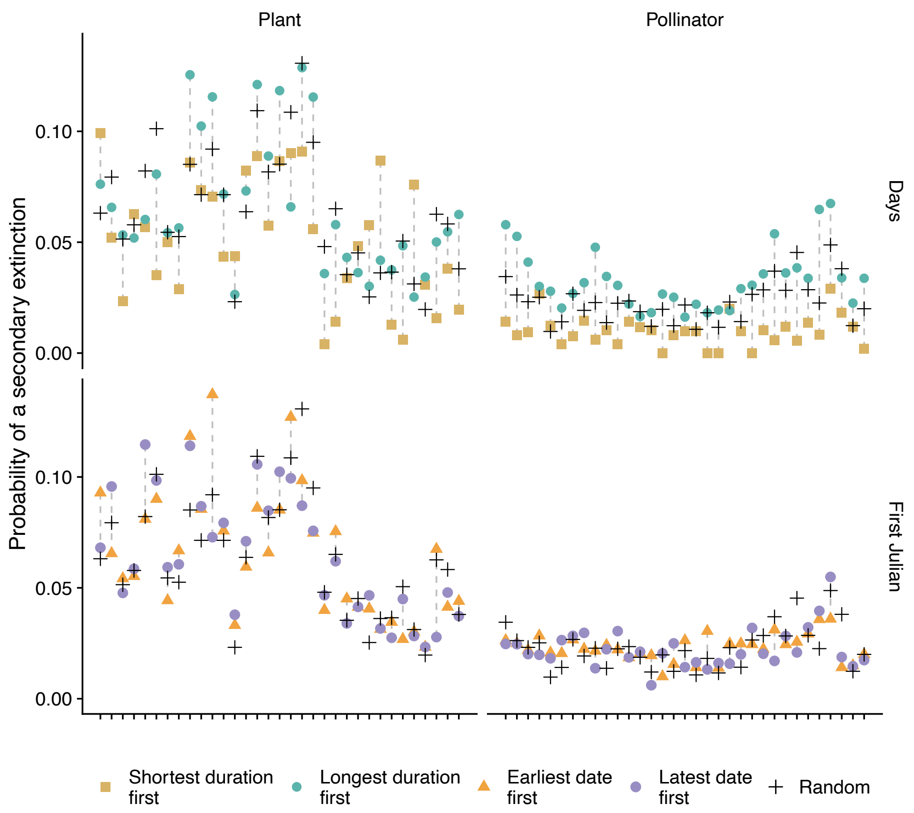

D) Extinction threshold = 0.25, Ri = 0.3- 0.6, distance based on the interaction matrix

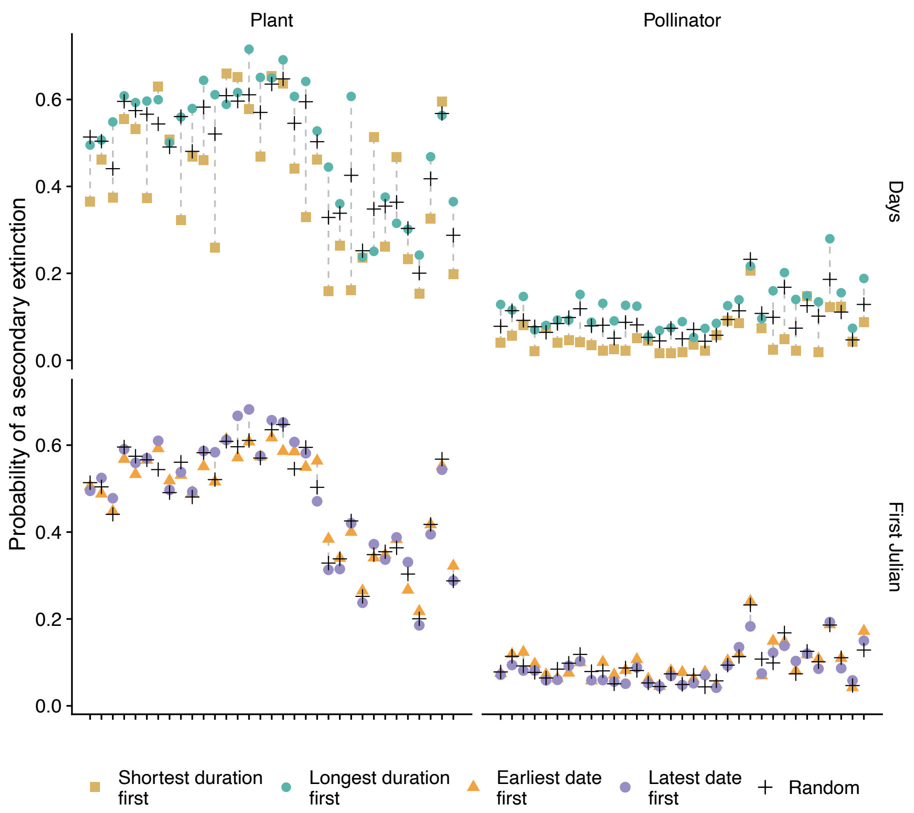

E) Extinction threshold = 0.5, Ri = 0.3- 0.6, distance based on the interaction matrix

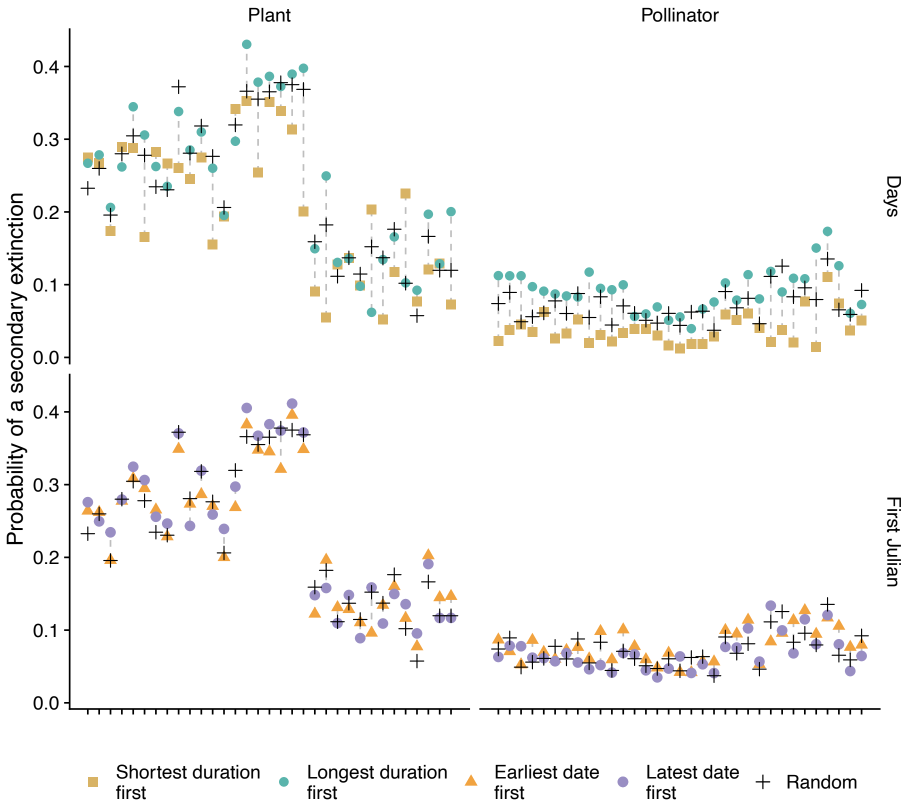

F) Extinction threshold = 0.75, Ri = 0.3- 0.6, distance based on the interaction matrix

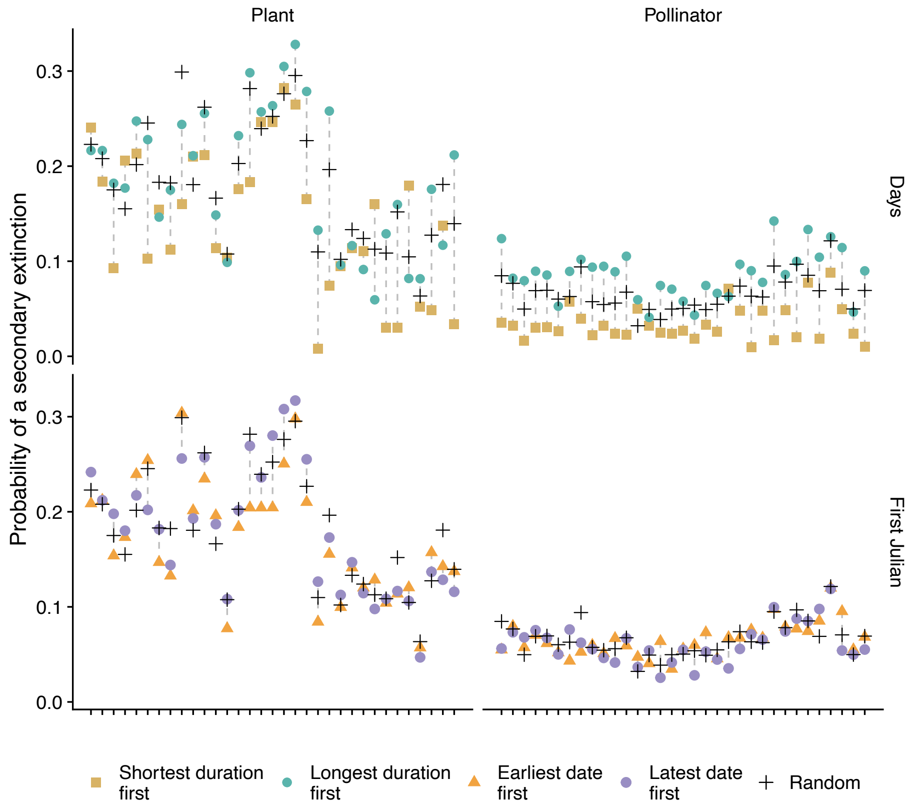

G) Extinction threshold = 0.25, Ri = 0.6- 0.9, distance based on the interaction matrix

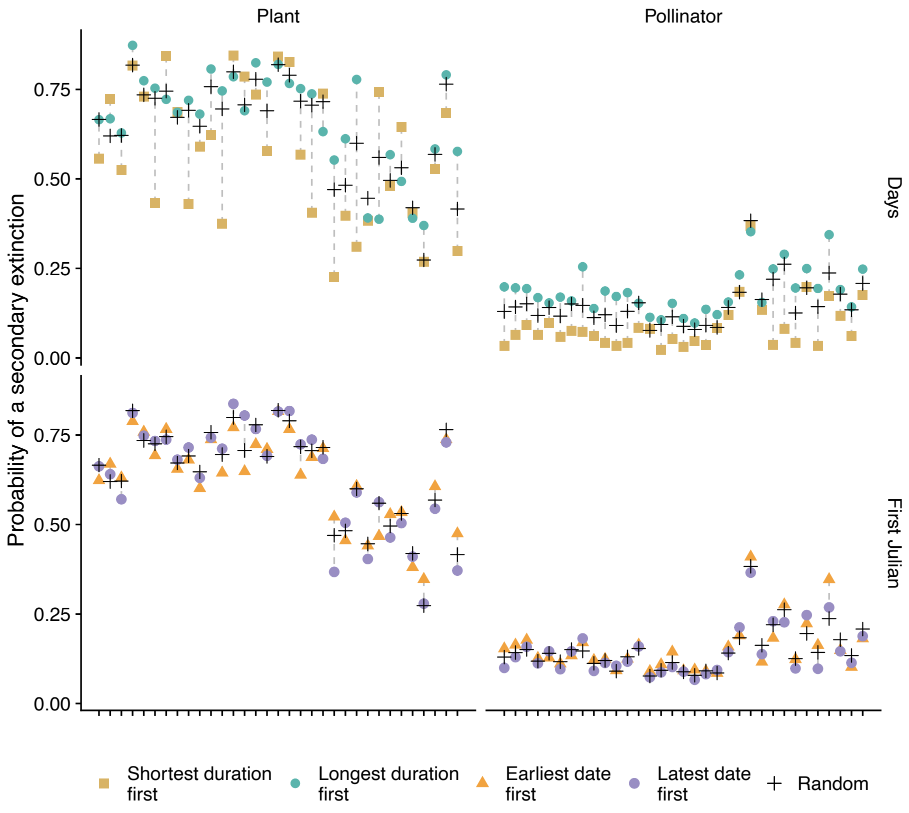

H) Extinction threshold = 0.5, Ri = 0.6- 0.9, distance based on the interaction matrix

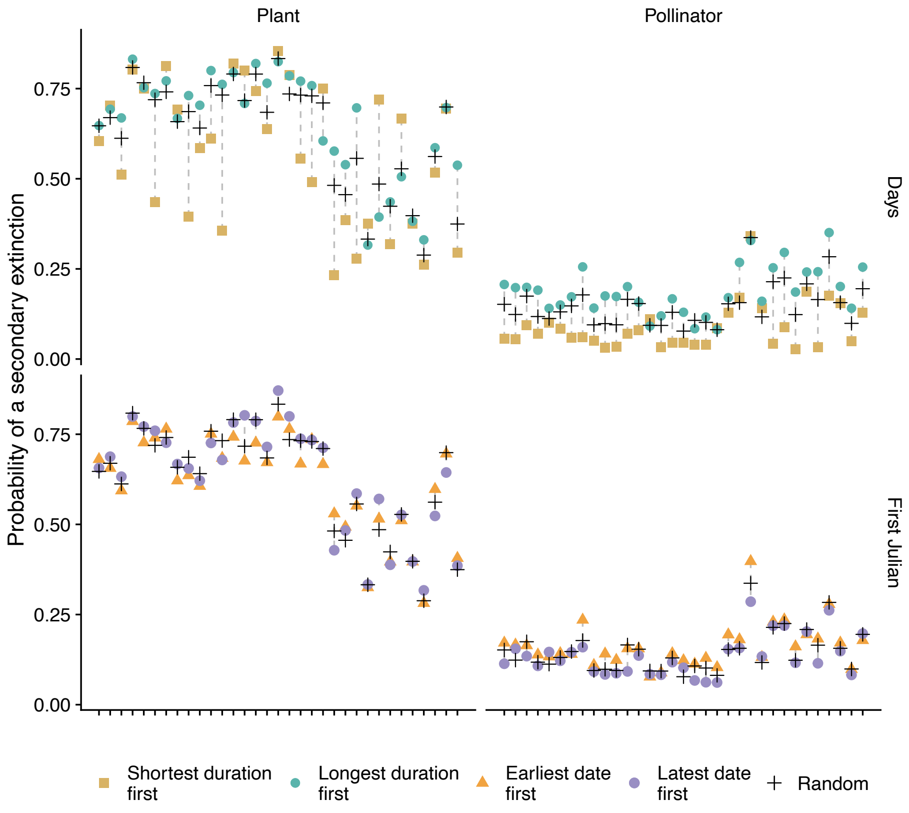

I)Extinction threshold = 0.75, Ri = 0.6- 0.9, distance based on the interaction matrix

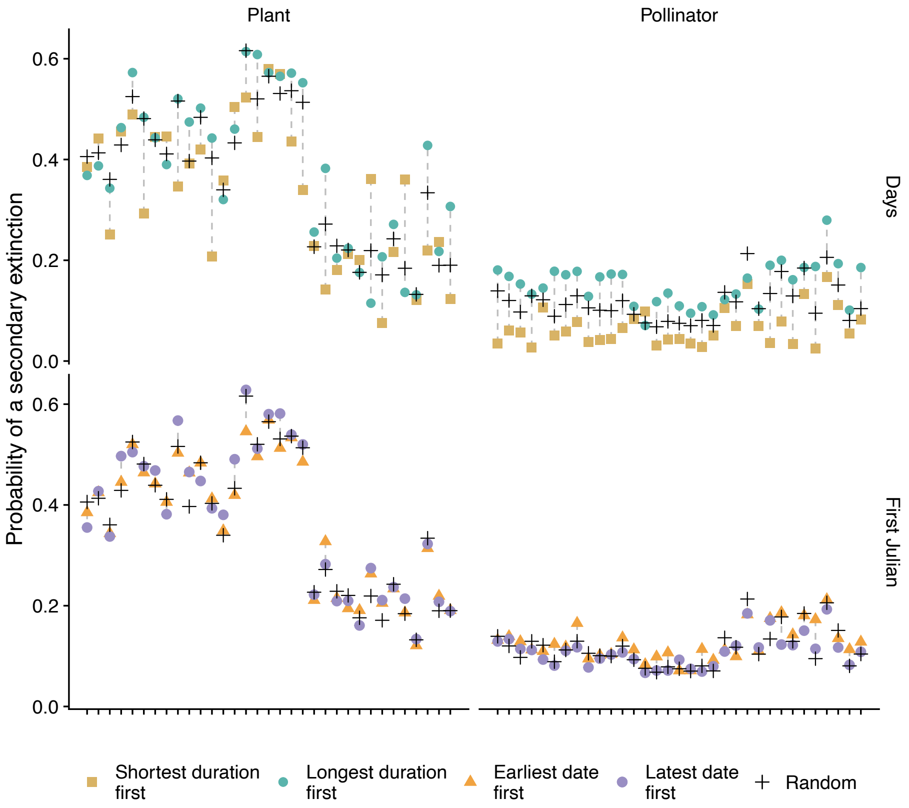

J) Extinction threshold = 0.25, Ri = 0- 0.3, distance based on phenological variables

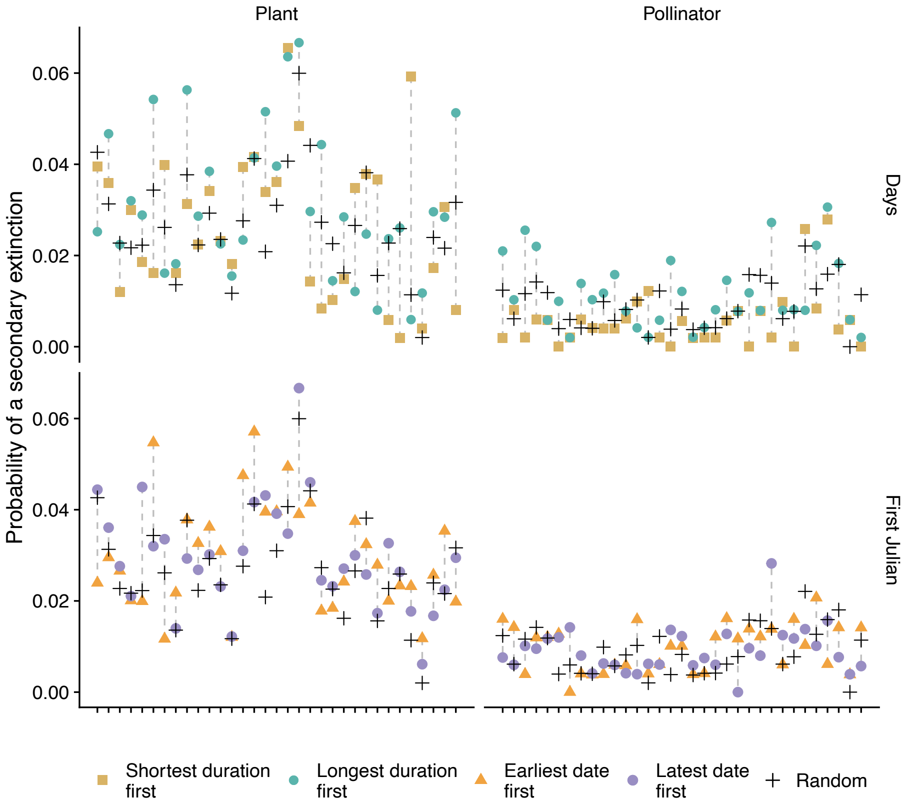

K) Extinction threshold = 0.5, Ri = 0- 0.3, distance based on phenological variables

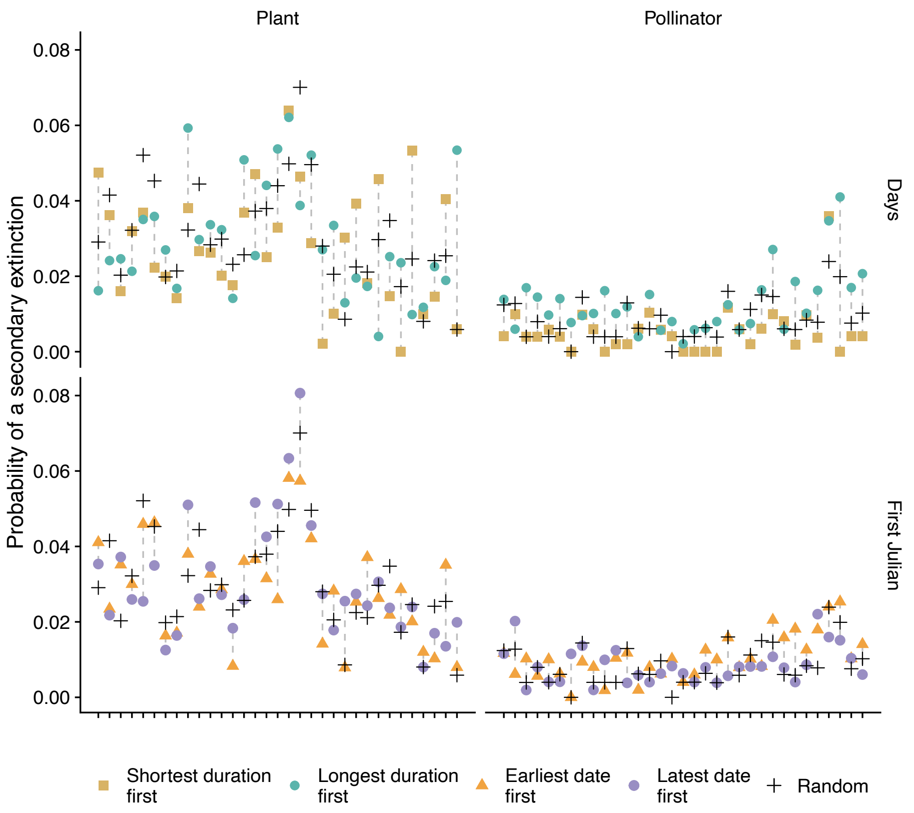

L) Extinction threshold = 0.75, Ri = 0- 0.3, distance based on phenological variables

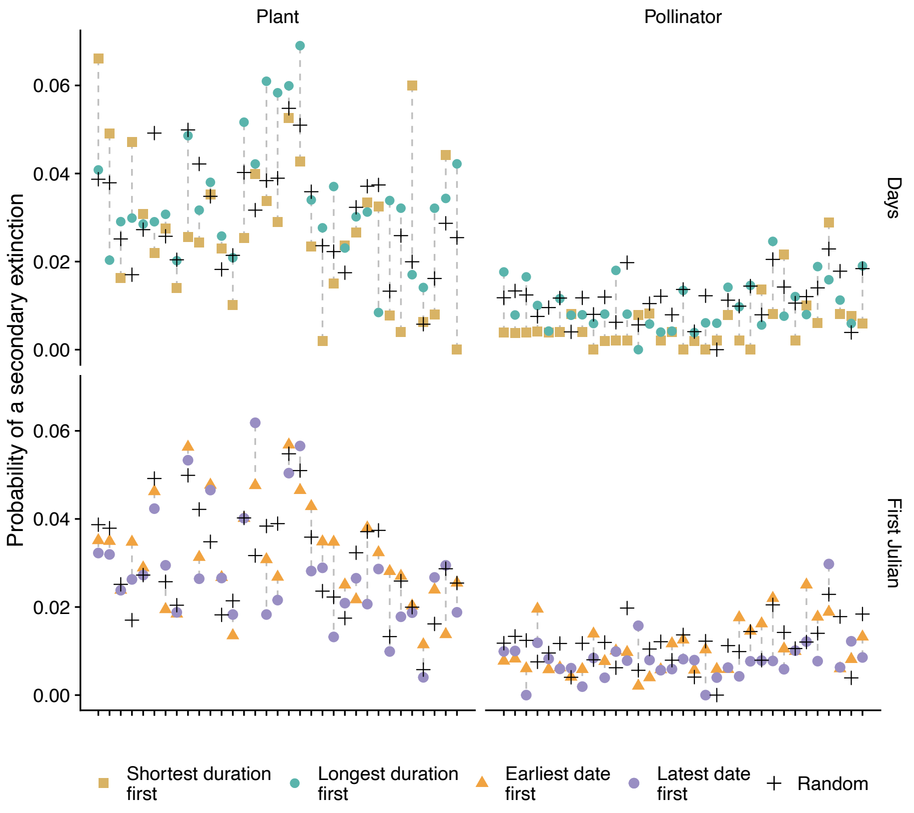

M) Extinction threshold = 0.25, Ri = 0.3 - 0.6, distance based on phenological variables

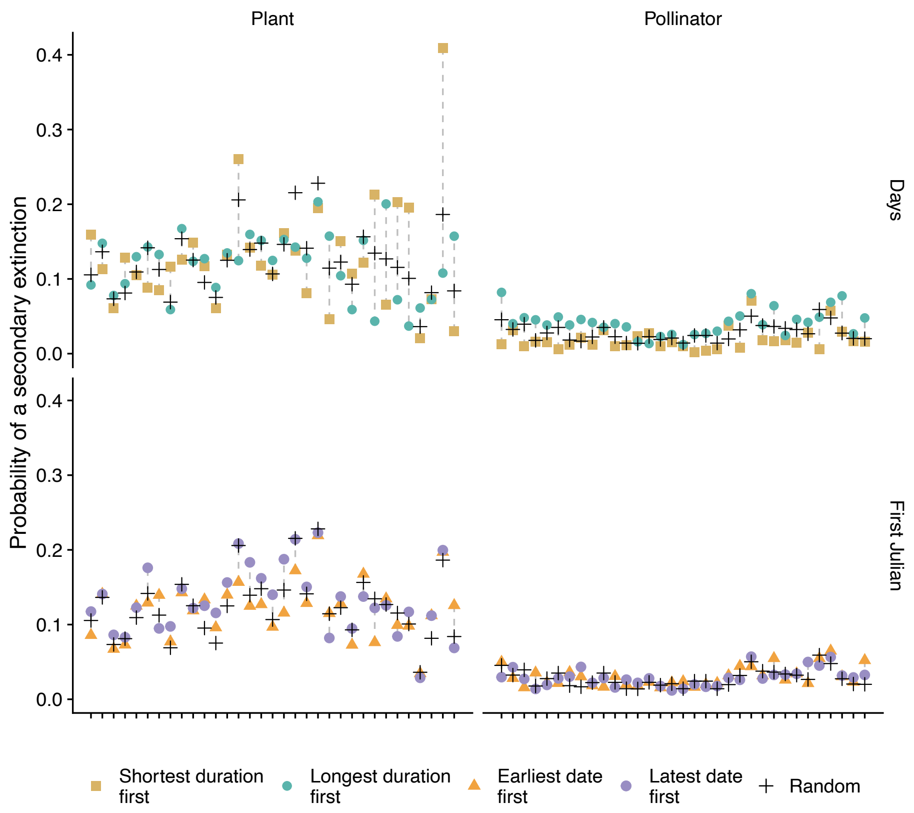

N) Extinction threshold = 0.5, Ri = 0.3 - 0.6, distance based on phenological variables

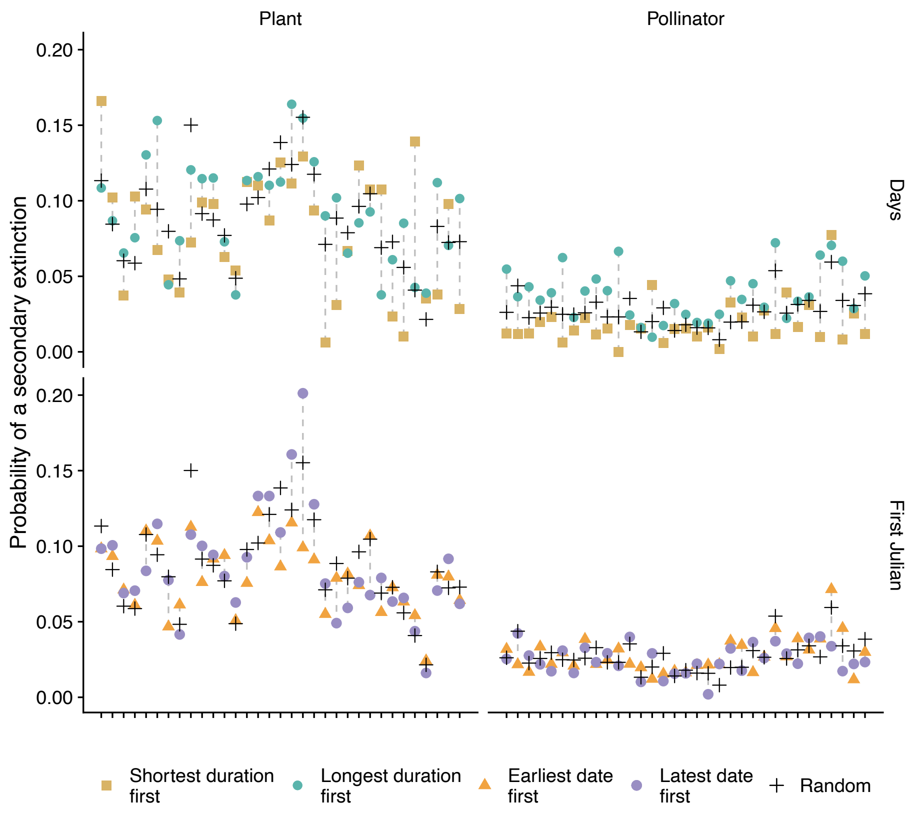

O) Extinction threshold = 0.75, Ri = 0.3 - 0.6, distance based on phenological variables

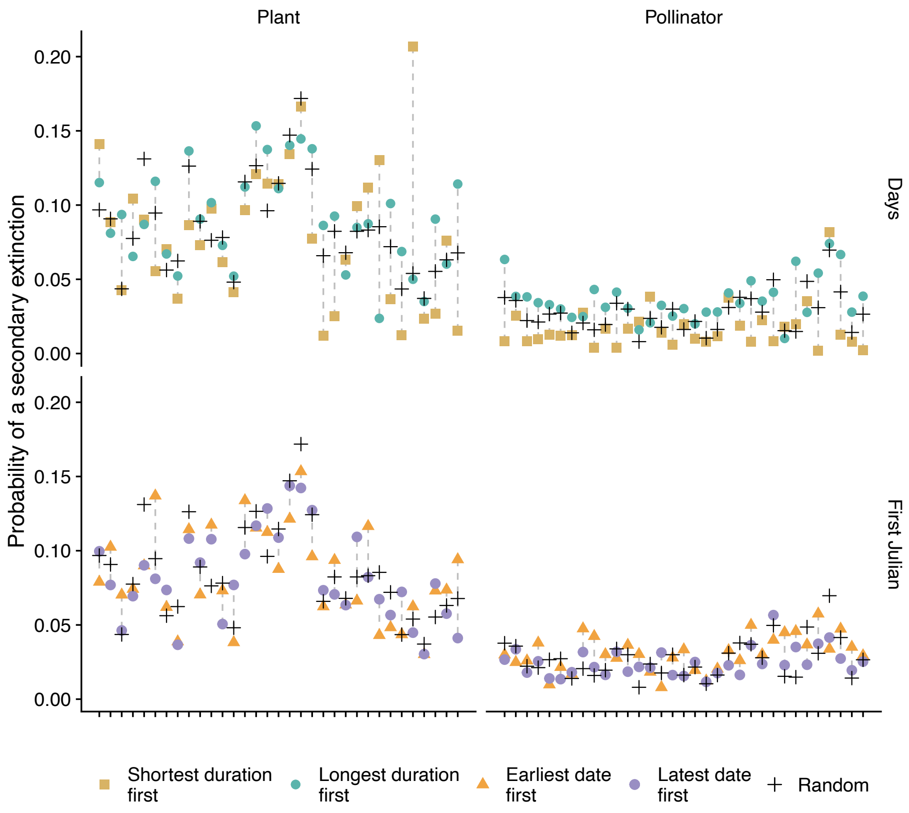

P) Extinction threshold = 0.25, Ri =0.6-0.9, distance based on phenologica variables

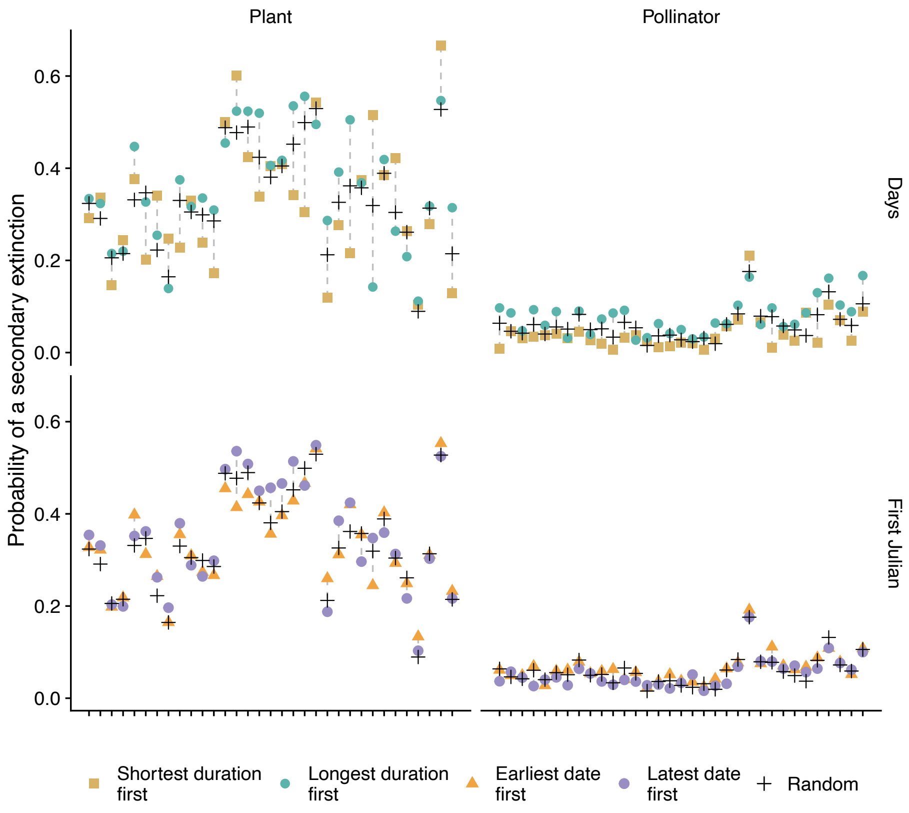

Q) Extinction threshold = 0.5, Ri = 0.6-0.9, distance based on phenological variables

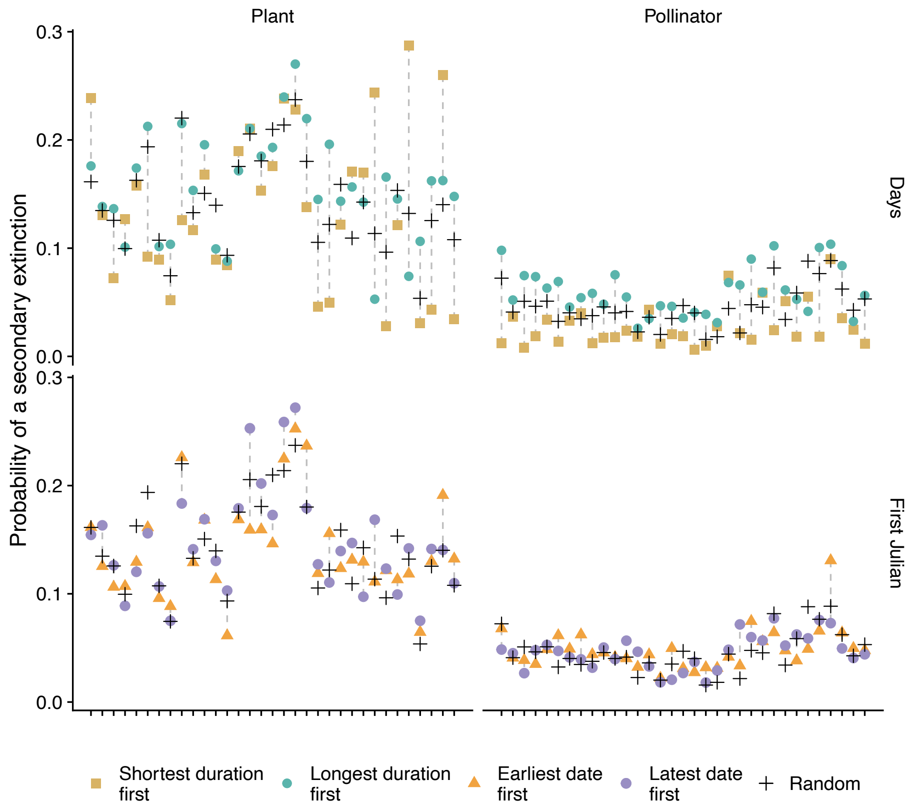

R) Extinction threshold = 0.75, Ri = 0.6-0.9, distance based on phenological variables

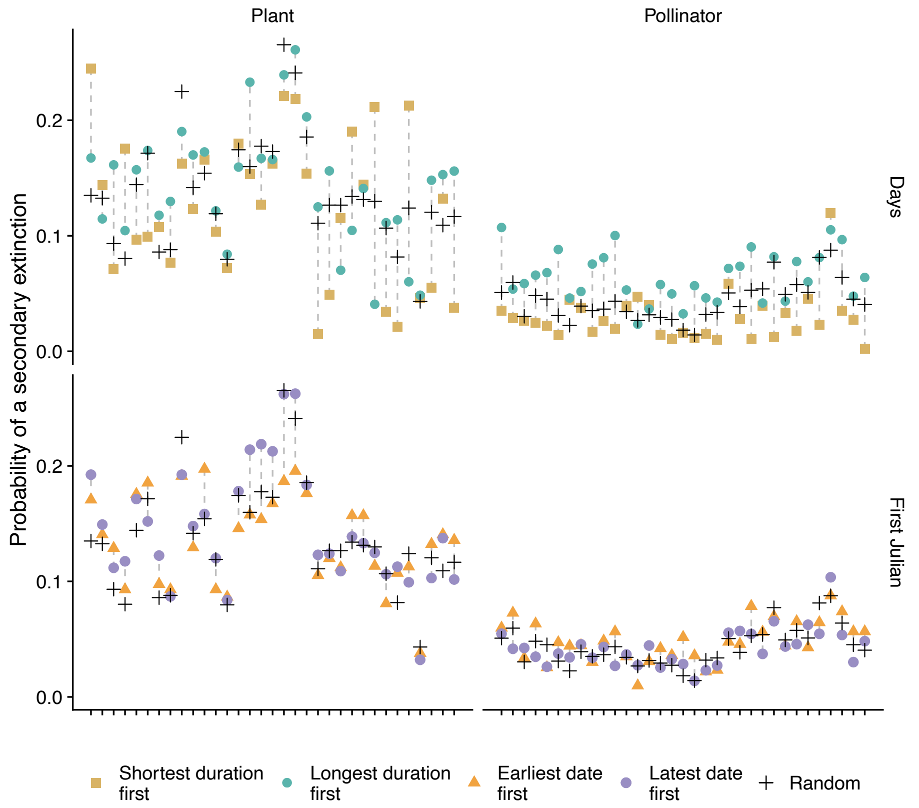

Supplement: Supplementary file 1 — Supplementary Material [file ECE3-11-13321-s001.pdf]
